# Ectopic Osteogenesis and Scaffold Biodegradation of Nano-Hydroxyapatite-Chitosan in a Rat Model

**DOI:** 10.1371/journal.pone.0135366

**Published:** 2015-08-10

**Authors:** Yiqun He, Youhai Dong, Fuzhai Cui, Xujun Chen, Rongqiang Lin

**Affiliations:** 1 The Orthopaedic Department of The Fifth People’s Hospital of Shanghai, Fudan University, Shanghai, China; 2 State Key Laboratory of New Ceramic and Fine Processing, Department of Materials Science and Engineering, Tsinghua University, Beijing, China; Institute for Frontier Medical Sciences, Kyoto University, JAPAN

## Abstract

The bone-formation and scaffold-biodegradation processes have not been fully characterized. This study aimed to determine the osteogenic ability of nHA-CS osteo-induced bone marrow mesenchymal stem cell (BMSC) composites and to explore the relationship between bone formation and scaffold biodegradation. The nHA-CS osteo-induced BMSC composites (nHA-CS+cells group) and the nHA-CS scaffolds (nHA-CS group) were implanted into the femoral spatium intermusculare of SD rats. At 2, 4, 6, 8, and 12 weeks post-implantation, the rat femurs were scanned using computerized tomography (CT), and the CT values of the implants were measured and comparatively analyzed. The implants were then harvested and subjected to hematoxylin and eosin (HE) and Masson's trichrome staining, and the percentages of bone area, scaffold area and collagen area were compared between the two groups. The CT values of the implants were higher in the nHA-CS+cells group than the nHA-CS group at the same time points (*P* < 0.05). Histological analysis revealed that *de novo* bone and collagen formation in the pores of the scaffolds gradually increased from 2 weeks post-implantation in both groups and that the scaffold gradually degraded as bone formation proceeded. However, more *de novo* bone and collagen formation and scaffold degradation occurred in the nHA-CS+cells group than in the nHA-CS group at the same time points (*P* < 0.05). In conclusion, nHA-CS osteo-induced BMSC composites are promising bone tissue engineering substitutes, and osteo-induced BMSCs can significantly enhance the osteogenic ability and play an active role in the degradation of nHA-CS scaffolds on par with bone formation.

## Introduction

Bone tissue engineering provides a promising way for bone regeneration. Numerous investigations have confirmed the feasibility of reconstructing bone tissue via tissue engineering[1, 2]. Tissue engineered scaffolds composed of calcium phosphates and natural biopolymers are widely used in bone defect repair. Hydroxyapatite (HA, Ca_10_(PO_4_)_6_(OH)_2_) is the primary inorganic component of natural bone and is a promising biomaterial due to its excellent biocompatibility, bioactivity and osteoconductivity[3]. However, the application of pure HA scaffolds in bone tissue engineering is limited by their hardness, fragility, and lack of fiexibility[4, 5].The fragility makes it impossible for HA to be used in load-bearing bone repair and substitute, and the HA powders can easily migrate from the implant site which may result in inappropriate ossification. Compared to pure HA, polymeric nanocomposites exhibit improvements in properties such as modulus, strength, and stiffness for bone repair[6]. Inorganic–organic composite materials are increasingly important due to the extraordinary properties arising from the synergism between the properties of their components [7]. Therefore, novel composites of HA and organic polymers that can compensate for the weak mechanical and physical properties of HA are of substantial interest. Chitosan (CS, (C_6_H_11_O_4_N)_n_) is an N-deacetylation product of chitin and a unique polysaccharide biopolymer that can be degraded in vivo by chitosanases and glucosaminidase to ultimately form glucose[8]. In addition to its biodegradability and nontoxicity, CS exhibits other relevant properties, including biocompatibility, antimicrobial activity and minimal foreign body reaction[9, 10].

HA-CS nanocomposites are excellent candidates for bone regeneration due to their biocompatibility and osteoconductivity[11, 12]. These nanocomposites promote bone regeneration by supporting the adhesion, proliferation and activation of the integrin-BMP/Smad signaling pathway of BMSCs[13]. The effects of the surface morphology, surface wettability, and size distribution of HA/CS composites with different CS contents on bone regeneration potential have been studied extensively[14]. The biodegradation characteristics of the scaffold are also critical for bone regeneration applications. SN Danilchenko et al.[15]examined the in vivo biodegradation behavior of simple chitosan/hydroxyapatite scaffolds inserted in a perforated tibial defect of rats, and found that the porous chitosan/hydroxyapatite composites gradually integrated into the newly formed bone matrix from the 10th day but were completely replaced by newly formed bone tissue by the 24th day during the course of bone remodeling. Sun T et al.[16] determined that an HA-CS scaffold degraded more rapidly than pure CS scaffolds in phosphate-buffered saline (PBS) in vitro, despite slower uptake properties. From the Danilchenko's study, scaffold biodegradation occurred concomitant with the bone-formation process. Bone marrow mesenchymal stem cells (BMSCs), a subset of nonhematopoietc cells in bone marrow stroma, can be easily isolated from a small aspirate of bone marrow and readily generate single-cell-derived colonies. These cells are extensively applied in tissue engineering for their rich source, low immunogenicity and multi-directional differentiation potency[17]. Based on these past results, we hypothesized that promoting bone formation by combining a scaffold with osteo-induced BMSCs would promote scaffold biodegradation on par with bone formation.

The objectives of this study were to investigate the biodegradability of nHA-CS scaffolds and to explore the relationship between bone formation and scaffold biodegradation with or without cells. After the nHA-CS scaffolds were prepared, the nHA-CS osteo-induced BMSCs composites and the pure scaffolds were intramuscularly implanted into Sprague-Dawley (SD) rats. Scaffold biodegradation and bone formation were analyzed, and the nHA-CS osteo-induced BMSC composites and the pure scaffolds were compared.

## Materials and Methods

### Experimental design

BMSCs isolated from bone marrow harvested from 8-week-old SD rats were expanded and osteogenically induced in vitro at the third passage. The tissue engineering grafts were constructed by seeding the osteo-induced BMSCs onto nHA-CS scaffolds. The prepared tissue engineering grafts (the experimental group, nHA-CS osteo-induced BMSCs composites) and the nHA-CS scaffolds (the control group) were separately implanted intramuscularly in the thighs of SD rats. Bone formation and scaffold biodegradation were evaluated by imaging and histology at 2, 4, 6, 8, and 12 weeks post-implantation. At each time point, six implants were harvested from each of the experimental and control groups.

### Preparation and characterization of scaffolds

The scaffolds were synthesized by a chemical co-precipitation method[18]. Briefly, CS powder (80% degree of deacetylation, Haidebei Bioengineering Co. Ltd, Jinan, China) was dissolved in dilute hydrochloric acid (0.1 mol/L) to obtain a 2% CS hydrochloride solution, which was then cooled to 4°C. Then, 1 g of nHA powder (particle sizes of 70–100 nm, Beijing DK nanotechnology Co. Ltd, Beijing, China) was added per 100 ml of CS hydrochloride solution to maintain a CS/nHA weight ratio of 2, and the composites were stirred until an adequate suspension was obtained. Subsequently, 1 ml of a solution of glycerophosphate (560 mg/ml), which was used as a cross-linking agent, was added to 10 ml of the suspension, followed by stirring for 30 min at 37°C. An nHA-CS hydrogel was then obtained. The hydrogels were carefully washed with distilled water to remove hydrochloric acid and excess glycerophosphate until the pH of the eluate was about 7, and freeze-dried at -20°C to obtain the nHA-CS scaffolds. The nHA-CS scaffolds were prepared as tablets with diameters of 14 mm and thickness of 3 mm and scanned using a scanning electron microscope (SU8010, Hitachi, Japan) to determine the average pore diameter and average porosity. All scaffolds were sterilized with ethylene oxide[19].The sterilizations were performed by exposure to Honeywell Oxyfume-30 (30% EtO and 70% CO_2_) for 8 h at 40°C and 40%–50% relative humidity. An initial vacuum of 400 mmHg (53.3 kPa) was applied for 15 min, and then 600 mg/L of Oxyfume-30 was added until the chamber reached a pressure of 0.5 kgf/cm^2^ (49.0 kPa). After the incubation period, a 400 mmHg vacuum was reestablished. The scaffolds were purged with N_2_ several times to remove residual EtO and stored at room temperature for at least 1 week before experiment.

### Isolation, expansion and osteogenic induction of BMSCs

All animal procedures were performed in strict accordance with the recommendations in the Guide for the Care and Use of Laboratory Animals of the National Institutes of Health. The protocol was approved by the Committee on the Ethics of Animal Experiments of Fudan University (Permit Number: 13–1428).The Percoll gradient centrifugation was applied in the BMSCs' isolation[1]. After preparation of the skin, a SD rat was sacrificed by cervical dislocation under general anesthesia using 10% chloral hydrate solution (0.4 ml/100 g). Under sterile conditions, the rat femurs were excised. The bone marrow was extracted by flushing the medullar cavities of the femurs with a basic culture medium consisting of LG-DMEM (Gibco BRL, Gaithersburg, MD, USA) supplemented with 10% fetal bovine serum (Gibco BRL, Gaithersburg, MD, USA), 100 U/ml penicillin and 100 μg/ml streptomycin (both from AMRESCO LLC, Solon, OH, USA) (Table 1). The marrow was then transferred into a 5 ml preheparinized centrifugation tube(Corning, Union City, CA, USA). Mononuclear cells were separated by Percoll (1.073 g/ml) gradient centrifugation (at 2000 r/min for 10 min) and seeded into 6-mm tissue culture dishes (Corning, Union City, CA, USA) at a density of 1×10^5^ cells/cm^2^ in basic culture medium in a 25 cm^2^ culture incubator(Corning, Union City, CA, USA) at 37°C and 5% CO_2_. The medium was changed on the third day to remove nonadherent cells and subsequently changed twice each week. Confluent BMSCs were detached using 0.25% trypsin/EDTA (Gibco BRL, Gaithersburg, MD, USA) and subcultured at a density of 1×10^5^ cells/cm^2^ in new tissue culture dishes. The BMSCs were subcultured until the third passage.

**Table 1:** The detailed basic culture medium component list.

components	concentration	manufactures' information
LG-DMEM	/	Gibco BRL, Gaithersburg, MD, USA
fetal bovine serum	volume fraction 10%	Gibco BRL, Gaithersburg, MD, USA
penicillin	100 U/ml	AMRESCO LLC, Solon, OH, USA
streptomycin	100 μg/ml	AMRESCO LLC, Solon, OH, USA

The third passage cells were exposed to osteogenic medium composed of basic culture medium, 10 mmol/l β-glycerophosphate, 10^−8^ mol/l dexamethasone and 50 mg/l L-ascorbic acid (all from Sigma, St. Louis, MO, USA) (Table 2)[1]. After induction in osteogenic medium for one week, the BMSCs were subjected to calcium-cobalt staining[20] to examine alkaline phosphatase (ALP) activity, and the calcium nodules deposited in the extracellular matrix were detected via Alizarin red[21] stain after the three-week induction.

**Table 2:** The detailed osteogenic medium component list.

components	concentration	manufactures' information
LG-DMEM	/	Gibco BRL, Gaithersburg, MD, USA
fetal bovine serum	volume fraction 10%	Gibco BRL, Gaithersburg, MD, USA
penicillin	100 U/ml	AMRESCO LLC, Solon, OH, USA
streptomycin	100 μg/ml	AMRESCO LLC, Solon, OH, USA
β-glycerophosphate	10 mmol/L	Sigma, St. Louis, MO, USA
dexamethasone	10^−8^ mol/L	Sigma, St. Louis, MO, USA
L-ascorbic acid	50 mg/L	Sigma, St. Louis, MO, USA

### Cell seeding and surgical procedure

Before cell seeding, the nHA-CS scaffolds were prewetted in basic culture medium for 4 h and dried in an incubator overnight after the medium was aspirated under sterile conditions. Two-week-old osteo-induced BMSCs were trypsinized and then resuspended in osteogenic medium at a density of 1×10^6^ cells/ml. Each side of the scaffold was seeded with 100 μl of the suspension and then incubated overnight at 37°C and 5% CO_2_ to permit cell attachment. After 12 h, six composites were washed with osteogenic medium, and the unattached cells in the medium were counted via manual haemocytometer. The average cell adhesion rates were calculated as the percentage of the attached cells versus seeded cell number to assess the cell-seeding efficacy of the nHA-CS scaffolds. In a parallel experiment, to visualize cell attachment on the scaffolds, the cells seeded on scaffolds were dyed with fluorescent 4’,6-diamidino-2-phenylindole (DAPI) for 15 min, followed by fluorescent inverted microscopy analysis. The composites were cultured in dishes in a mobile incubator with the temperature of 37°C in transit.

The recommendation and approval of the animal procedures were the same with those stated in the BMSCs' isolation section. All surgery was performed using chloral hydrate anesthesia, and all efforts were made to minimize suffering. Thirty-two-month-old male SD rats underwent the same surgical procedure using experimental animal operation tables. Briefly, the animals were placed under general anesthesia using 10% chloral hydrate solution (0.4 ml/100 g) via intraperitoneal injection and placed in a lateral position. After preparation of the skin, a 1-cm-long incision was made along the femoral axis, and the femoral muscle was bluntly dissected, exposing the spatium intermusculare used in this model. The cells/scaffold composites or scaffolds were implanted into the femoral spatium intermusculare, and the incisions were sutured. Postoperatively, thirty rats implanted on the left side with nHA-CS scaffolds (the nHA-CS group) and on the right side with cell/nHA-CS composites (the nHA-CS+cells group) were fed as usual and housed with five rats per cage. The conditions of the rat were monitored, cages were cleaned, with water and food ad libitum.

### Computerized tomography (CT) evaluation

The rats were anesthetized at 2, 4, 6, 8, and 12 weeks after implantation via intraperitoneal injection with a 10% chloral hydrate solution (0.4 ml/100 g), and their thighs were then scanned along the axial plane by computerized tomography (CT, Light Speed 64 slice spiral CT, General Electric, USA, 2.5 mm slice thickness). The position and bone formation in the implants were observed in the CT images, and simultaneously, CT values of the newly formed bone were measured and comparatively analyzed.

### Histological analysis

After CT scanning, the rats were sacrificed via cervical vertebra dislocation under anesthesia, and the implants were harvested, fixed in 4% paraformaldehyde (pH 7.26) for 24 h, decalcified in 10% EDTA for 2 to 7 days, and embedded in paraffin. The histological stain was conducted by a professional pathologist, and the histological review was conducted by another independent reviewer. Serial 5-μm-thick sections were prepared and stained with hematoxylin and eosin (HE), and 6 parts in the bone forming area were selected randomly under the microscope as the target total area. The amount of newly formed bone was assessed as a percentage of the bone area versus the total area and the amount of the remaining scaffold was assessed as a percentage of the scaffold area versus the total area by measuring the area on the HE-stained images using Image-Pro Plus software. Masson’s trichrome staining of the implant sections was also performed to quantify the collagen secreted in the process of bone formation as an indirect index of the amount of newly formed bone[22].

### Statistical analysis

All data were collected with n = 6 and are expressed as mean ± standard deviation (SD). A 2-factor ANOVA and Student’s paired *t*-test using SPSS 16.0 were applied to determine possible significant differences. A *P* value ≤ 0.05 was considered significant.

## Results

### Characterization of the nHA-CS scaffolds

**Fig 1:**
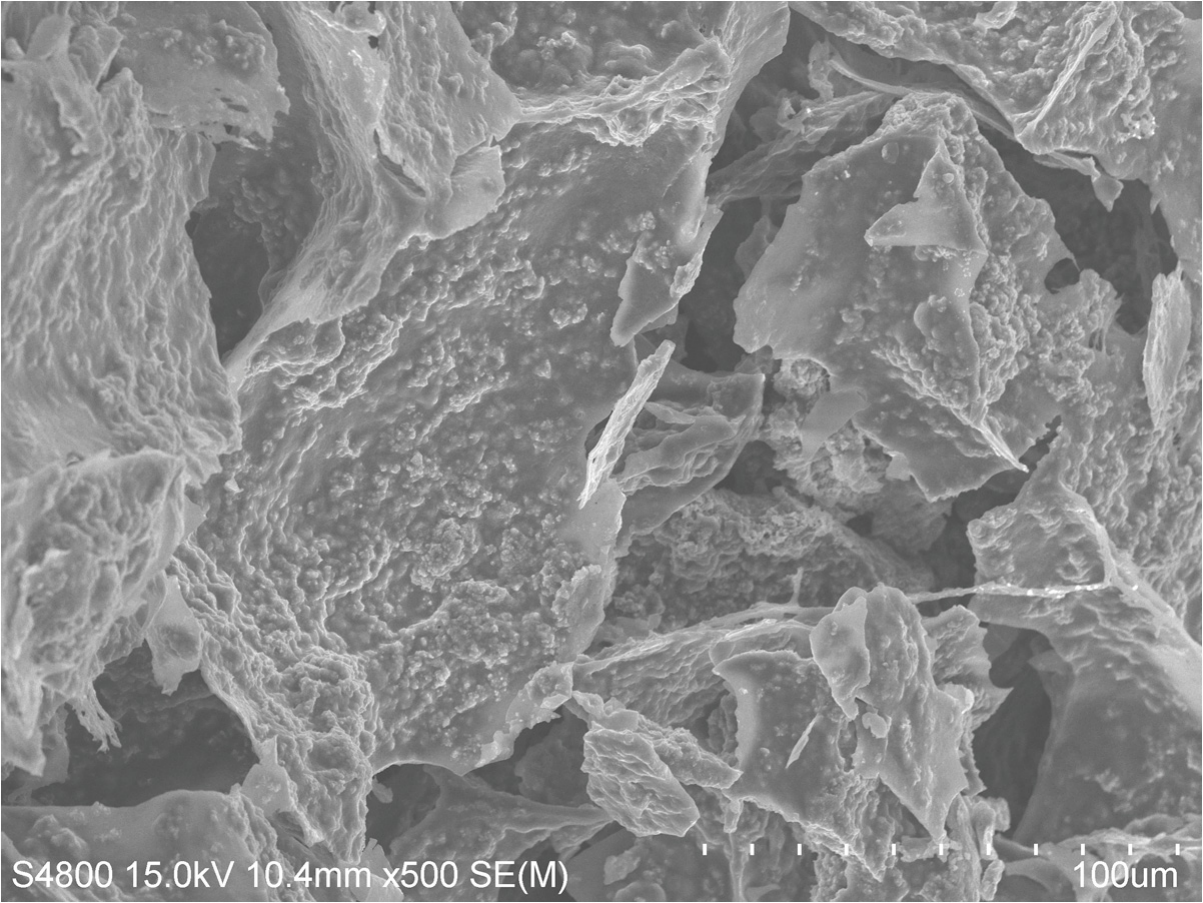
Micrographs of nHA-CS. The nHA-CS scaffold exhibited a three-dimensional porous structure in which the nHA particles were evenly distributed throughout the pore surface (figure magnification 500×).

Electron microscopy revealed that the nHA-CS scaffolds exhibited a three-dimensional porous structure, with nHA particles evenly distributed in the pore surface of the nHA-CS scaffolds (Fig 1). The mean pore diameter of the nHA-CS scaffolds was (583.45 ± 167.15) μm, and the mean porosity of the nHA-CS scaffolds was (88.43 ± 1.74)%.

### Identification of osteogenically induced BMSCs

**Fig 2:**
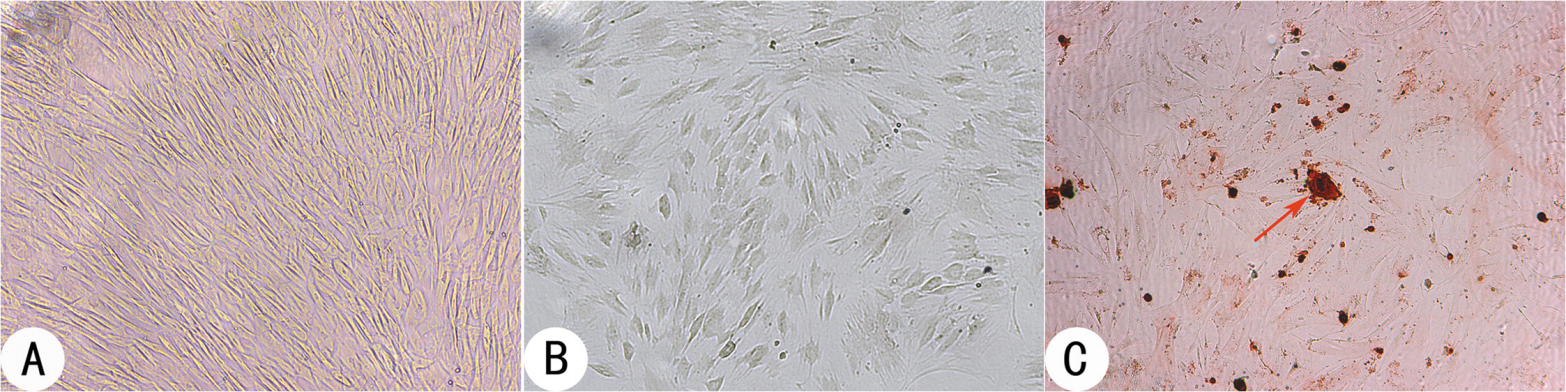
BMSCs and identification of osteogenic differentiation at passage 3. **(A)** BMSCs cultured in basic medium exhibited typical elongated fibroblast-like morphology. **(B)** ALP expression was identified by calcium-cobalt staining of black particle deposition in cells after one week of osteogenic induction. **(C)** Calcified nodules that formed in the extracellular matrix were detected by Alizarin red staining after three weeks of osteogenic induction. The red arrow indicates a calcified nodule (figure magnification 100×).

BMSCs cultured in basic media exhibited the elongated fibroblast-like shape typical of third-passage morphology (Fig 2A). After induction in osteogenic media for one week, the BMSCs exhibited a morphological conversion to the cuboidal osteoblast-like shape, and ALP expression was detected by calcium-cobalt staining (Fig 2B). Alizarin red staining indicated extracellular matrix mineralization and calcified nodule formation after the BMSCs were induced for three weeks (Fig 2C).

### Cell seeding efficacy and attachment on the scaffolds

**Fig 3:**
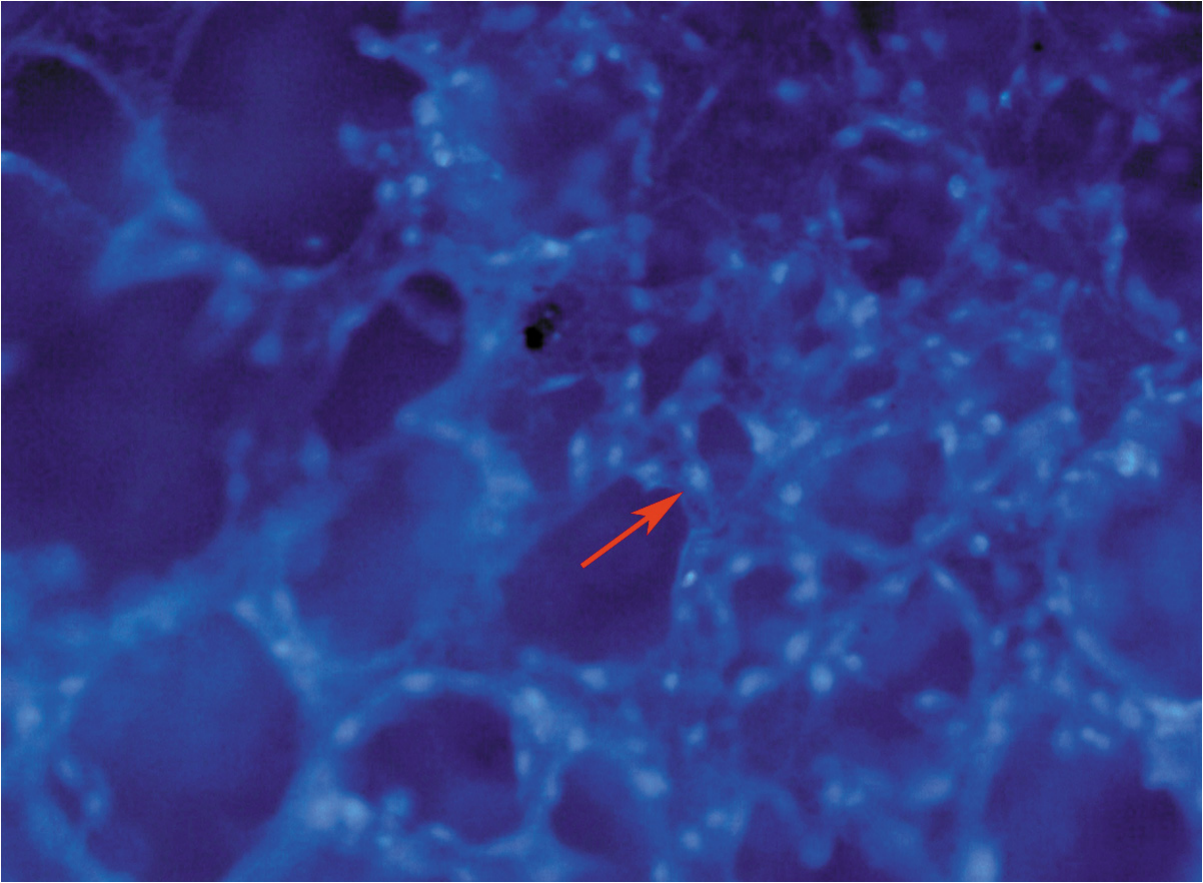
Fluorescent image of DAPI-labeled cells on pore surfaces of scaffolds. BMSCs adhered in the vicinity of the pores and its edges of a nHA-CS scaffold. Red arrows indicate labeled cells (figure magnification 100×).

The mean osteogenically induced BMSC adhesion rate of the nHA-CS scaffolds was (88.02 ± 2.07)% (n = 6). Cells attached to the nHA-CS scaffolds were observed using CLSM. As shown in Fig 3, numerous labeled BMSCs adhered in the vicinity of the pores and its edges.

### CT evaluation

**Fig 4:**
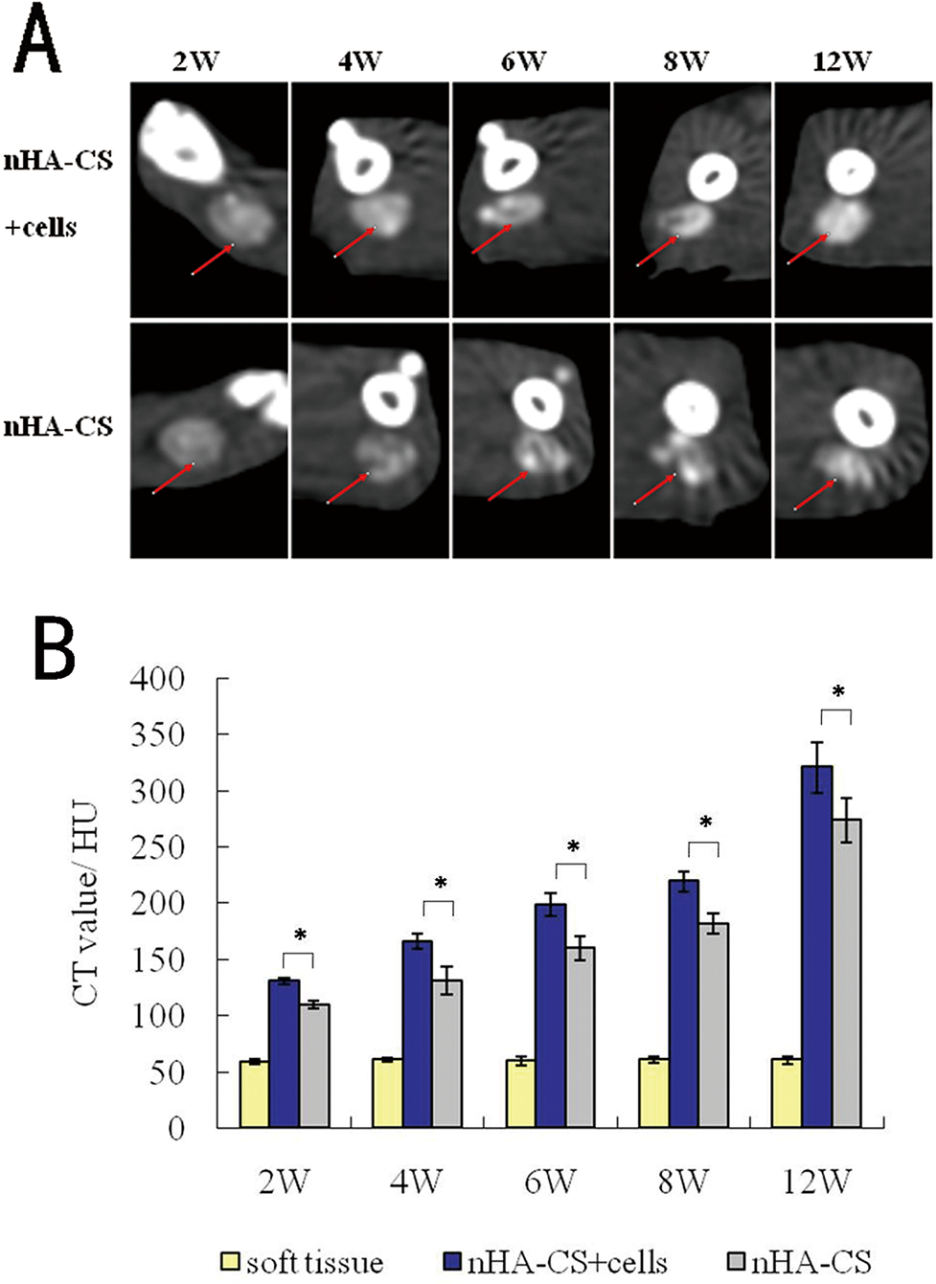
CT evaluation of the implants. **(A)** Representative CT images of the implants scanned at 2, 4, 6, 8, and 12 weeks post-implantation. Red arrows indicate the implants. **(B)** The CT values of the implants in the nHA-CS+cells group were significantly greater than those of the nHA-CS group (*P < 0.05), indicating that *de novo* bone formation was greater in the nHA-CS+cells group.

To determine the location of new bone formation within the implants, rat femurs were scanned by CT at 2, 4, 6, 8, and 12 weeks post-implantation, and representative images from each group are shown in Fig 4A. In both groups, the implants were located in the femoral spatium intermusculare, and new bone formation could be observed at 2 weeks post-implantation. The CT values of the implants in the nHA-CS group were (109.42±3.42) Hu, (130.81±12.25) Hu, (160.36±10.84) Hu, (181.80±9.23) Hu and (273.78±19.35) Hu at 2, 4, 6, 8 and 12 weeks post-implantation, and those in the nHA-CS+cells group were comparatively (131.06±2.68) Hu, (166.18±6.64) Hu, (198.83±9.91) Hu, (219.50±9.16) Hu and (320.65±22.69) Hu. A tendency toward an increase in newly formed bone was observed in both groups. The statistical analysis of the CT values of each group also demonstrated that *de novo* bone gradually increased with time. The CT values of implants in the nHA-CS+cells group were 17%-27% more than those in the nHA-CS group, which demonstrated that the nHA-CS+cells group formed significantly more *de novo* bone than the nHA-CS group (*P< 0.05, Fig 4B).

### Histological analysis

**Fig 5:**
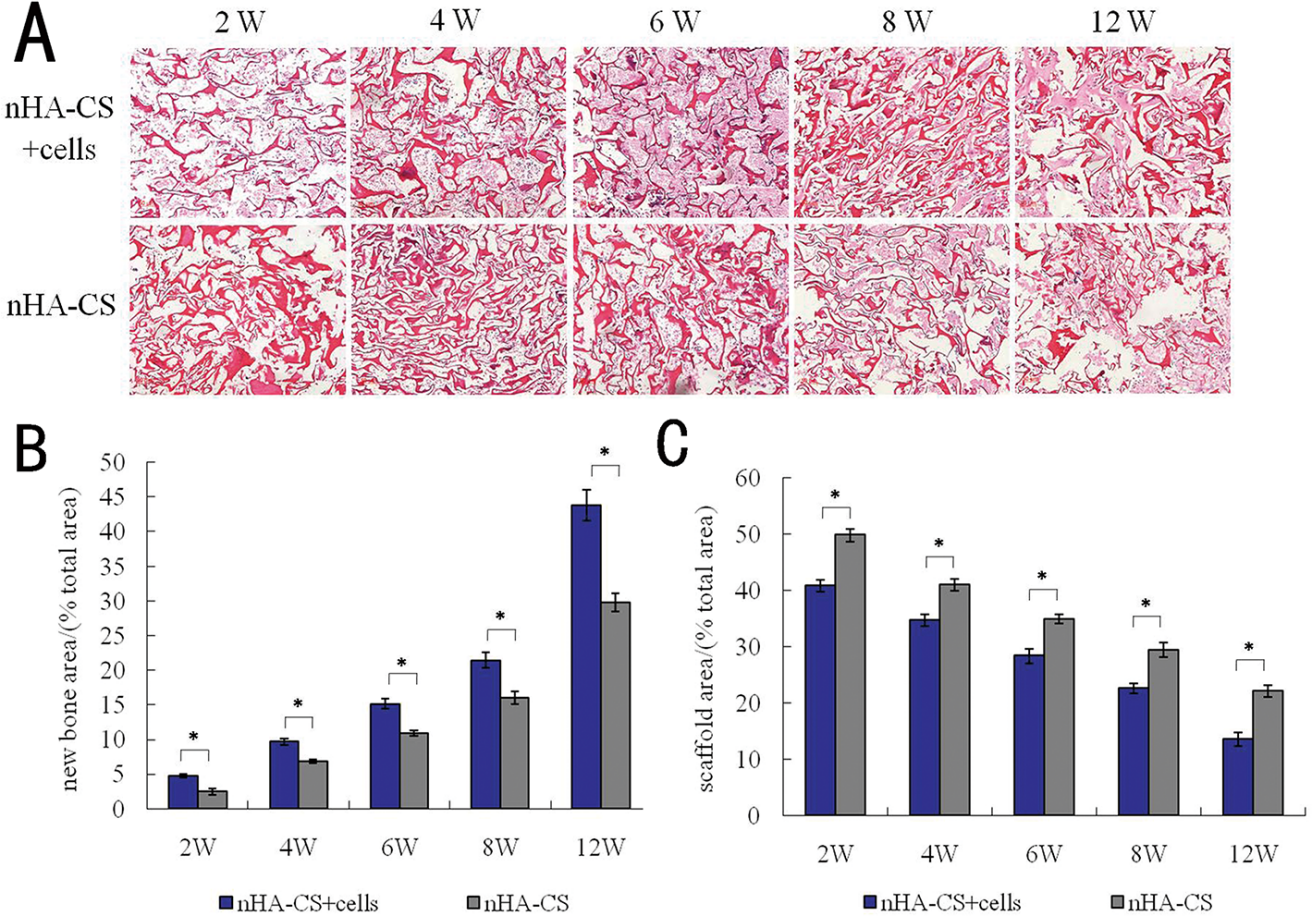
Histological examination of HE staining. **(A)** Representative HE images of implants from the two groups harvested at different time points (figure magnification 100×). **(B)** The analysis of the proportions of newly formed bone area indicated that the nHA-CS+cells group gained greater amounts of *de novo* bone compared with the nHA-CS group (*P < 0.05). **(C)** The analysis of the proportions of the scaffold area revealed that the nHA-CS+cells group exhibited significant scaffold degradation than the nHA-CS group (*P < 0.05).

The *de novo* bone was visualized by HE staining of each group at each time point. When the nHA-CS scaffolds that were combined with the osteogenically induced BMSCs were implanted intramuscularly into SD rats, medullary bone tissue formed in the scaffold pores at week 2 post-implantation. Osteogenesis subsequently continued to develop and extend throughout the scaffolds, and medullary bone deposition gradually increased to fill the scaffold pores at week 4 post-implantation. As the osteogenic process progressed, some of the medullary bone connected to form woven bone and chondroid bone at week 6 post-implantation, which continued until week 8. Prior to week 12, most of the *de novo* bone was woven bone or chondroid bone. By contrast, in the nHA-CS group, only medullary bone deposition occurred until week 6, and the woven bone formed at week 8 (Fig 5A). The proportions of bone area in the nHA-CS group were (2.46±0.41) %, (6.88±0.28) %, (10.95±0.41) %, (15.97±0.93) % and (29.78±1.34) % at 2, 4, 6, 8 and 12 weeks post-implantation, and those in the nHA-CS+cells group were comparatively (4.76±0.29) %, (9.65±0.50) %, (15.14±0.69) %, (21.45±1.13) % and (43.80±2.25) %.The proportions of bone area in the nHA-CS+cells group were 34%-93% more than those in the nHA-CS group, which demonstrated that larger amount of *de novo* bone was formed in the nHA-CS+cells group compared to the nHA-CS group (*P < 0.05, Fig 5B).

As shown in Fig 5A, the nHA-CS scaffold gradually degraded over time. In the nHA-CS+cells group, the thickness of the pore walls gradually decreased, and the pore diameters gradually increased after week 2 post-implantation. The breakdown of the pore walls could be observed at week 6 and was very obvious at week 12, when the scaffold visibly disintegrated into pieces. A similar process of scaffold biodegradation was observed in the nHA-CS group. The proportions of scaffold area in the nHA-CS group were (49.76±1.14) %, (40.90±1.04) %, (34.91±0.80) %, (29.42±1.28) % and (22.07±1.03) % at 2, 4, 6, 8 and 12 weeks post-implantation, and those in the nHA-CS+cells group were comparatively (40.76±1.02) %, (34.65±1.05) %, (28.33±1.26) %, (22.55±0.89) % and (13.58±1.21) %.The proportions of scaffold area remaining were 15%-38%lower in the nHA-CS+cells group than the nHA-CS group(*P < 0.05, Fig 5C), indicating that the scaffold degraded more rapidly in the nHA-CS+cells group.

**Fig 6:**
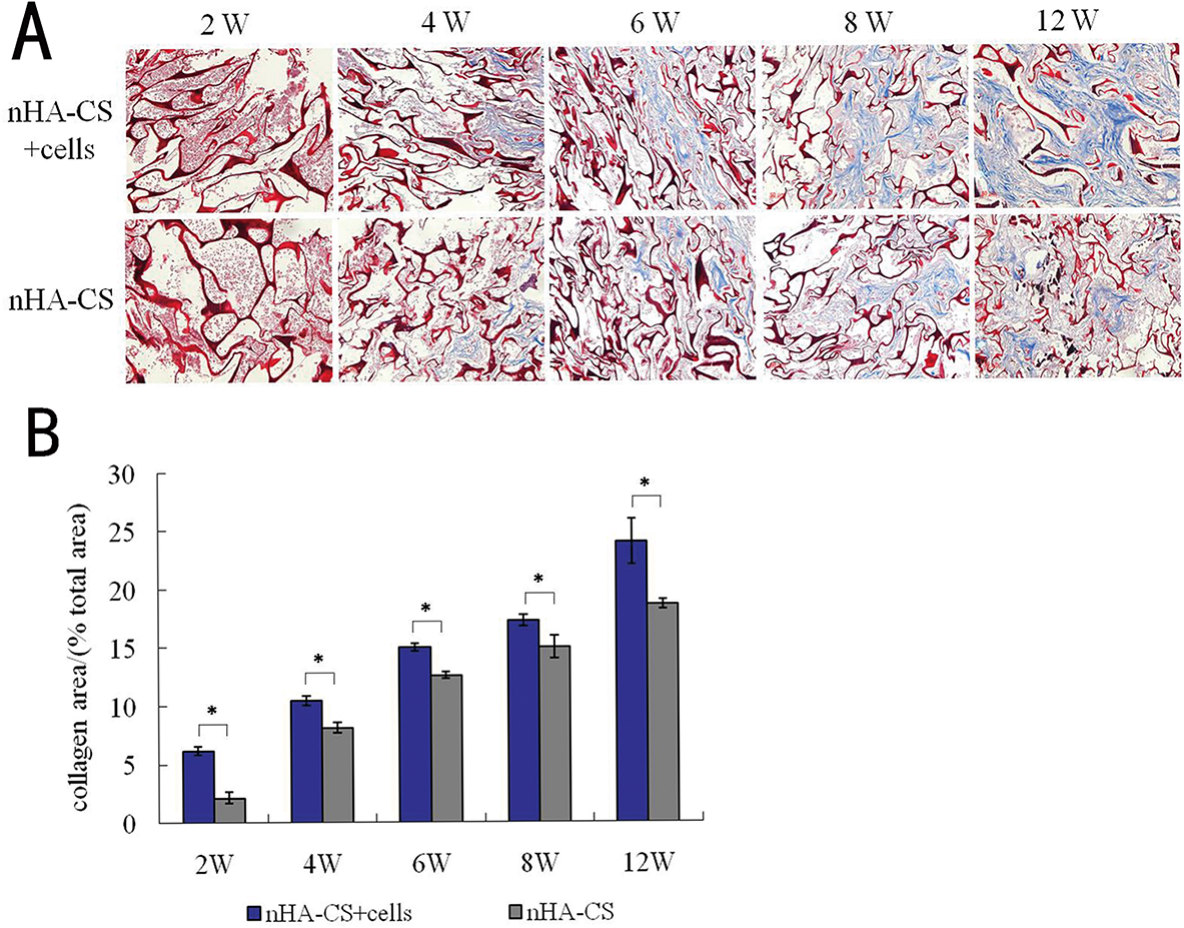
Histological examination of Masson's trichrome staining. **(A)** Representative Masson's trichrome staining images showing the expression of collagen in the two groups at different time points (figure magnification 100×). **(B)** The analysis of the proportions of the collagen area indicate that significantly more collagen was present in the nHA-CS+cells group than the nHA-CS group (*P < 0.05), indicating that the nHA-CS+cells group formed more *de novo* bone than the nHA-CS+cells group.

Masson's trichrome staining allowed the expression of collagen to be visualized as blue-stained tissue in both groups from week 2 to week 12. In the medullary bone-depositing stage, collagen was secreted to form collagen fibers along with the bone deposition at week 2, and the collagen fibers subsequently connected to create collagen nets to form the basic framework for bone formation at week 4. With the increase in secreted collagen, collagen fibers gradually merged to form the tabular structure as the mineralization matrix from week 6 (Fig 6A). The proportions of collagen area in the nHA-CS group were(2.15±0.48) %, (8.14±0.43) %, (12.61±0.27) %, (15.01±0.97) % and (18.68±0.44) % at 2, 4, 6, 8 and 12 weeks post-implantation, and those in the nHA-CS+cells group were comparatively (6.18±0.36) %, (10.50±0.42) %, (15.01±0.32) %, (17.32±0.51) % and (24.05±1.97) %. As the most important component of bone tissue, collagen progressively increased with bone formation, and the discrepancy between the two groups revealed that the nHA-CS+cells group produced 15%-187% more collagen than the nHA-CS group, which indirectly indicates that bone formation was increased and more rapid in the nHA-CS+cells group (*P< 0.05, Fig 6B).

## Discussion

Engineering scaffolds for bone tissue repair and regeneration must consider biodegradability, cell biology, biomolecules and the mechanical properties of the scaffold material[23]. Composite scaffolds based on a biodegradable natural polymer and osteoconductive hydroxyapatite (HA) nanoparticles are promising for a variety of tissue engineering applications. HA-CS nanocomposites are excellent materials for local bone regeneration due to their biocompatibility, osteoconductivity and biodegradability[11, 12, 16]. The biodegradation of HA-CS nanocomposites appears to occur on the same time scale as the bone formation process[15]. However, the relationship between scaffold biodegradation and bone formation has not been studied in detail.

In this study, bone formation and scaffold biodegradation processes based on nHA-CS osteo-induced BMSC composites and pure scaffolds were compared to identify whether promoting the bone formation would catalyze scaffold degradation. After the scaffold/cell composites and pure scaffolds were implanted into the femoral spatium intermusculare of SD rats (the nHA-CS+cells group and the nHA-CS group, respectively), *de novo* bone formation and scaffold degradation were observed in the pore regions from week 2 to week 12 post-implantation. In both groups, the amount of *de novo* bone gradually increased and the remaining scaffold gradually decreased with time. At each time point, more *de novo* bone formation and, accordingly, less scaffold remnants were observed in the nHA-CS+cells group compared to the nHA-CS group.

We observed two trends in bone formation. The first trend was a progressive increase in bone formation in each group with time. And Ye X et al[24] observed the similar trend in their comparative research in ectopic bone regeneration by combining beta-tricalcium phosphate scaffold with human bone marrow mononucleated cells, undifferentiated and osteogenically differentiated bone marrow mesenchymal stem cells. However, until week 12 post-implantation, no evidence of bone remodeling and lamellar bone was observed, which was different from their work. The reason most likely lied in the lack of mechanical stimulation. Mechanical stimulation, either from the scaffolds or from the implanted host, might enhance cell osteogenic activity and osteoinductivity[25, 26] as well as stimulate bone formation and remodeling[27, 28]. The second trend was that *de novo* bone formation was greater in the nHA-CS+cells group than the nHA-CS group, which indicates that pre-osteogenically induced BMSCs can significantly promote local bone formation. Ye X et al[24] also demonstrated that osteogenically differentiated BMSCs could significantly enhance the bone forming ability of the composites. This result demonstrates that the composites composed of nHA-CS scaffolds and osteo-induced BMSCs were superior to nHA-CS scaffolds for supporting bone regeneration. Yu B et al[29] evaluated the ectopic bone formation with or without BMSCs of an injectable chitosan/hydroxyapatite/collagen (CS/nHAC) composites, and demonstrated that the BMSCs seeded CS/nHAC composites were superior to CS/nHAC composites in ectopic bone formation.

Hematoxylin and eosin (HE) staining revealed that the proportion of the scaffold area gradually decreased with time in both groups. At each time point, less scaffold material remained in the nHA-CS+cells group than the nHA-CS group, confirming that osteo-induced BMSCs promoted the biodegradation of the scaffold. However, combined analysis of bone formation and scaffold biodegradation revealed that the *de novo* bone and scaffold area remained nearly constant in the two groups and did not increase with time, indicating that the biodegradation rate was positively correlated with the speed of bone formation. Sun T et al[16] confirmed that the HA-CS scaffold would lose its 38% weight after degrading in pure phosphate buffered sodium (PBS) for 4 weeks. In our work, the proportions of nHA-CS scaffold area dropped by approximately 67% after degrading in vivo for 12 weeks with BMSCs, and 56% without BMSCs. Scaffold biodegradation can be accelerated by free radicals and superoxide anions produced by the cellular medium and enzymes triggered by the inflammatory response to the muscle injury resulting from exogenous implants[30]. Reactive oxygen species (ROS), which are key mediators of cell function in both health and disease, particularly at sites of inflammation and tissue healing, play an important role in triggering cell-mediated scaffold degradation[31]. Moreover, the synergism of osteogenesis and vascularization may promote scaffold biodegradation via local recruitment of giant cells to erode and absorb the scaffold and increase the blood supply to clear the metabolic products[32]. We inferred that the scaffold biodegradation was primarily due to exposure to the local inflammatory response to the muscle injury and the exogenous implant, which was promoted by the synergism of osteogenesis and vascularization. Therefore, although the rate of scaffold degradation corresponded with the rate of bone formation in both groups, the ability of the osteo-induced BMSCs to promote osteogenesis indirectly enhanced scaffold biodegradation in the nHA-CS+cells group through synergism of osteogenesis and vascularization. We also inferred that scaffold biodegradation may be promoted by the local enrichment of macrophages, which could be triggered by the expression of chemokines produced by osteo-induced BMSCs or inflammatory cells during bone formation. However, the precise mechanism of scaffold biodegradation in vivo remains unknown and will be studied in our future work.

Type I collagen, which is primarily synthesized by differentiated osteoblasts and comprises 90% of the organic matrix of bone, fabricates the major protein framework of bone as a template for matrix mineralization to sustain the integrity and flexibility of bone[33]. Therefore, collagen expression was analyzed by Masson’s trichrome staining as an indirect index of *de novo* bone formation[34]. In this study, collagen expression followed the same trend observed for bone formation by HE staining, indicating the excellent bone inductivity and conductivity of the nHA-CS scaffold/cell composites. Song SH et al[35] stained collagen by Masson’s trichrome staining to investigate the bone regeneration in rat tibial defect, and similar to our work, the collagen staining showed the same trend with the HE staining.

During the process of bone formation, bone density gradually increases as a result of local calcium deposition and mineralization, resulting in increased CT values of the implants [36]. Progressive increase in bone density was observed in the CT images for each group, and the CT values revealed a gradual increase in calcium deposition and mineralization. Similar results were reported in Yu B's work that the CT values of implants would increase along with time and were higher in the scaffold with cells group than in the pure scaffold group[29].The significant difference between the nHA-CS+cells group and the nHA-CS group further demonstrates the advantage of cells/scaffold composites over pure scaffolds. Besides, an interesting progression of bone formation from outside to inside was observed on the CT images, indicating the importance of blood supply. This centripetal ossification sequence closely related to the vascular ingrowth process[37]; the supply of blood and the presence of pre-induced cells synergistically improve bone formation[38, 39].

BMSCs have been applied extensively in bone tissue engineering techniques due to their unlimited supply, multilineage potential and self-renewal capability[40] and are frequently subject to osteogenic induction prior to application. Although factors promoting osteogenic induction have been identified, such as TGF-β, BMP, curcumin and mechanical stimulation[25, 26, 41, 42], the classical osteogenic medium is basic medium supplemented with 10 mM β-glycerophosphate, 10^−8^ M dexamethasone and 50 mg/l L-ascorbic acid. To determine the effect of osteogenic induction on the BMSCs, calcium-cobalt staining and Alizarin red staining were performed. In vitro osteogenic differentiation of the BMSCs was confirmed by the high levels of ALP expression and extracellular calcium deposition, which indicate that delivering a mature osteoblastic population contributes to more rapid and extensive bone formation in vivo.

## Conclusions

In this study, the osteogenic performance and biodegradation of the nHA-CS scaffold with or without the osteogenically induced BMSCs were compared using a SD rat femoral spatium intermusculare model. The results demonstrated that the nHA-CS scaffold/cell composites triggered more significant *de novo* bone formation and scaffold degradation than the pure scaffolds. Moreover, the biodegradation of nHA-CS scaffolds corresponds to the pace of bone formation, and thus, nHA-CS scaffolds are promising material for bone regeneration.

## Supporting Information

S1 Table The CT values of the implants in the nHA-CS group and the nHA-CS+cells group (Hu, mean±SD). (DOCX)

S2 Table The proportions of bone area in the nHA-CS group and the nHA-CS+cells group (%, mean±SD). (DOCX)

S3 Table The proportions of scaffold area in the nHA-CS group and the nHA-CS+cells group (%, mean±SD). (DOCX)

S4 Table The proportions of collagen area in the nHA-CS group and the nHA-CS+cells group (%, mean±SD). (DOCX)
