# Supplementary material for: Ectopic Osteogenesis and Scaffold Biodegradation of Nano-Hydroxyapatite-Chitosan in a Rat Model
Source: PLoS One. 2015 Aug 10;10(8):e0135366. doi: 10.1371/journal.pone.0135366 (PMC4530870; doi:10.1371/journal.pone.0135366)
Supplement: S1 Table — (DOCX) [file pone.0135366.s001.docx]

S1 Table The CT values of the implants in the nHA-CS group and the nHA-CS+cells group (Hu, mean±SD)

|  | 2 weeks | 4 weeks | 6 weeks | 8 weeks | 12 weeks | *P* |
| --- | --- | --- | --- | --- | --- | --- |
| nHA-CS | 109.42±3.42 | 130.81±12.25 | 160.36±10.84 | 181.80±9.23 | 273.78±19.35 | <0.001 |
| nHA-CS+cells | 131.06±2.68 | 166.18±6.64 | 198.83±9.91 | 219.50±9.16 | 320.65±22.69 | <0.001 |
| t | 12.197 | 6.218 | 6.414 | 6.947 | 3.849 |  |
| *P* | <0.001 | <0.001 | <0.001 | <0.001 | 0.003 |  |
