# Supplementary material for: Ectopic Osteogenesis and Scaffold Biodegradation of Nano-Hydroxyapatite-Chitosan in a Rat Model
Source: PLoS One. 2015 Aug 10;10(8):e0135366. doi: 10.1371/journal.pone.0135366 (PMC4530870; doi:10.1371/journal.pone.0135366)
Supplement: S2 Table — (DOCX) [file pone.0135366.s002.docx]

S2 Table The proportions of bone area in the nHA-CS group and the nHA-CS+cells group (%, mean±SD)

|  | 2 weeks | 4 weeks | 6 weeks | 8 weeks | 12 weeks | *P* |
| --- | --- | --- | --- | --- | --- | --- |
| nHA-CS | 2.46±0.41 | 6.88±0.28 | 10.95±0.41 | 15.97±0.93 | 29.78±1.34 | <0.001 |
| nHA-CS+cells | 4.76±0.29 | 9.65±0.50 | 15.14±0.69 | 21.45±1.13 | 43.80±2.25 | <0.001 |
| t | 11.141 | 11.799 | 12.721 | 9.161 | 13.105 |  |
| *P* | <0.001 | <0.001 | <0.001 | <0.001 | <0.001 |  |
