# Supplementary material for: Ectopic Osteogenesis and Scaffold Biodegradation of Nano-Hydroxyapatite-Chitosan in a Rat Model
Source: PLoS One. 2015 Aug 10;10(8):e0135366. doi: 10.1371/journal.pone.0135366 (PMC4530870; doi:10.1371/journal.pone.0135366)
Supplement: S3 Table — (DOCX) [file pone.0135366.s003.docx]

S3 Table The proportions of scaffold area in the nHA-CS group and the nHA-CS+cells group (%, mean±SD)

|  | 2 weeks | 4 weeks | 6 weeks | 8 weeks | 12 weeks | *P* |
| --- | --- | --- | --- | --- | --- | --- |
| nHA-CS | 49.76±1.14 | 40.90±1.04 | 34.91±0.80 | 29.42±1.28 | 22.07±1.03 | <0.001 |
| nHA-CS+cells | 40.76±1.02 | 34.65±1.05 | 28.33±1.26 | 22.55±0.89 | 13.58±1.21 | <0.001 |
| t | 14.467 | 10.372 | 10.766 | 10.781 | 13.113 |  |
| *P* | <0.001 | <0.001 | <0.001 | <0.001 | <0.001 |  |
