# Supplementary material for: Ectopic Osteogenesis and Scaffold Biodegradation of Nano-Hydroxyapatite-Chitosan in a Rat Model
Source: PLoS One. 2015 Aug 10;10(8):e0135366. doi: 10.1371/journal.pone.0135366 (PMC4530870; doi:10.1371/journal.pone.0135366)
Supplement: S4 Table — (DOCX) [file pone.0135366.s004.docx]

S4 Table The proportions of collagen area in the nHA-CS group and the nHA-CS+cells group (%, mean±SD)

|  | 2 weeks | 4 weeks | 6 weeks | 8 weeks | 12 weeks | *P* | |
| --- | --- | --- | --- | --- | --- | --- | --- |
| nHA-CS | 2.15±0.48 | 8.14±0.43 | 12.61±0.27 | 15.01±0.97 | 18.68±0.44 | <0.001 |  |
| nHA-CS+cells | 6.18±0.36 | 10.50±0.42 | 15.01±0.32 | 17.32±0.51 | 24.05±1.97 | <0.001 |  |
| t | 16.366 | 9.562 | 13.837 | 5.117 | 6.520 |  |  |
| *P* | <0.001 | <0.001 | <0.001 | <0.001 | <0.001 |  |  |
